# Sensor-Based Indices for the Prediction and Monitoring of Anterior Cruciate Ligament Injury: Reliability Analysis and a Case Study in Basketball

**DOI:** 10.3390/s21165341

**Published:** 2021-08-07

**Authors:** Luca Molinaro, Juri Taborri, Adriano Santospagnuolo, Mario Vetrano, Maria Chiara Vulpiani, Stefano Rossi

**Affiliations:** 1 Department of Economics, Engineering, Society and Business Organization (DEIM), University of Tuscia, 01100 Viterbo, Italy; luca.molinaro@unitus.it (L.M.); juri.taborri@unitus.it (J.T.); 2 Physical Medicine and Rehabilitation Unit, Sant ‘Andrea Hospital, “Sapienza” University of Rome, 00189 Rome, Italy; adrianosantospagnuolo1@hotmail.it (A.S.); mario.vetrano@uniroma1.it (M.V.); 3 Sports Medicine Institute CONI Rome, 00197 Rome, Italy; mariachiara.vulpiani@uniroma1.it

**Keywords:** sensor-based indices, reliability, anterior cruciate ligament, basketball, injury

## Abstract

The possibility of measuring predictive factors to discriminate athletes at higher risk of anterior cruciate ligament (ACL) injury still represents an open research question. We performed an observational study with thirteen female basketball players who performed monopodalic jumps and single-leg squat tests. One of them suffered from an ACL injury after the first test session. Data gathered from twelve participants, who did not suffer from ACL injury, were used for a reliability analysis. Parameters related to leg stability, load absorption capability and leg mobility showed good-to-excellent reliability. Path length, root mean square of the acceleration and leg angle with respect to the vertical axis revealed themselves as possible predictive factors to identify athletes at higher risk. Results confirm that six months after reconstruction represents the correct time for these athletes to return to playing. Furthermore, the training of leg mobility and load absorption capability could allow athletes to reduce the probability of new injuries.

## 1. Introduction

In recent years, the introduction of sensor-based protocols as screening tools for the identification of reliable metrics aimed at assessing the risk of injury in élite players has become necessary [1]. Among others, the injury of the knee anterior cruciate ligament (ACL) is the most problematic and widespread in contact sports. A long period of absence from playing, a high risk of re-injury and the difficulty of recovering the ability to express the same intensity and skills prior to the injury make it an injury feared by all athletes [2,3,4]. One of the most critical movements that causes the occurrence of an ACL injury is the landing phase after a jump [5,6,7,8,9]. When the landing phase is characterized by a short duration, a player must dampen the force of impact on the ground and, at the same time, they must release additional force to perform other movements, such as a change in direction and/or another upward jump. It is worth noting that previous studies have indicated that the most likely period for ACL injury occurrence is from 30 ms to 100 ms after contact time with the ground after a jump [10,11].

Several studies have analyzed the landing phase after a jump for the purposes of evaluation, rehabilitation and performance to identify risk factors in athletes and non-athletic, anamnestic and clinical subjects, as well as to establish the most suitable training and intervention strategies to prevent injuries in people at risk [11,12,13]. Video analysis represents the most widely used methodology for the identification of these risk factors. Among others, Landis et al. [14], when performing video analysis on data gathered from 180 athletes of different sports, suggested that the investigation of knee abduction at the landing time after a drop jump test reveals useful information about ACL injury risks. A further study from Numata et al. [15] confirmed how knee valgus during the landing phase of a drop jump is a potential risk factor for non-contact ACL injuries in female high school athletes. Though video analysis is useful, the use of optoelectronic systems and force platforms are even more popular in this context, since they also gather information related to knee kinetics and ground reaction forces, which have been demonstrated to be indices correlated to ACL injury risk [16,17,18]. Myer et al. [16] showed that an analysis of knee kinetics at the landing time could be an important method for ACL injury prevention; in fact, they found that female athletes at higher risk of injury were associated with an anomalous increment in the knee abduction moment. A study by Mokhtarzadeh et al. [17] demonstrated the antagonistic and agonistic roles of the Gastrocnemius and Soleus in ACL loading; Podraza et al. [18] pointed out that the relative interaction of all involved thigh and lower leg muscles during the landing phase should be considered when interpreting non-contact ACL injury mechanisms. However, the use of optoelectronic systems and force platforms is also related to a set of non-negligible limitations. First, analysis often requires time consuming subject preparation and data processing, resulting in the tricky application of this approach when used as a screening tool during training sessions. Second, such systems often require a large investment due to their financial costs. 

For these reasons, the last decade has been characterized by a continuous increase in wearable solutions designed by various practitioners [19]. Among others, inertial-based wearable sensors have become prevalent [20,21,22]. Janssen et al. [20] used a set of inertial sensors to obtain athlete kinematics upon landing after a jump, and found that the valgus excursion can be considered as a predictive factor; a similar approach has been proposed by Dowling et al. [21], who suggested an analysis of knee flexion angle after a jump in order to obtain useful information regarding knee stability. Taborri et al. [22] showed that data gathered from an inertial sensor when performing monopodalic jumps and single-leg squats are useful for a feed machine-learning algorithm that was able to identify athletes at higher risk injury. 

It is worth noting that the reliability of all the indices proposed in previous studies has not been tested, even though the metrological characteristic of reliability is recognized as fundamental to introduce objective indices into clinical routines [23]. In addition, to the best of our knowledge, all previous studies focused on the analysis of data related to a single jump only at the instant in which the feet reached the ground, without considering either the behavior during the stabilization period after touching the floor or the effects induced by repeated jumps. However, it is apparent that 50% of acute knee injuries are the result of suboptimal landing, indicating the importance of a proper landing and stabilization technique; in fact, an optimal landing technique involves a soft landing with active muscular control, where energy absorption is more efficient, thus reducing the load in lower extremities [24]. In addition, previous studies analyzed neither parameters related to leg stability nor other technical gestures regarding the jump, with the exception of a preliminary study proposed by the same authors of this paper [25].

Among others, basketball represents one of the main sports associated with a high rate of ACL injury due to the requirements for developing high forces in a short space of time, as well as constant contact between players [26,27,28]. In addition, women have a greater predisposition to ACL injury than men, with an injury rate ranging from 2 to 8 times higher due to anatomical, physiological and biomechanical factors [10,29,30]. From this perspective, this study’s aims are twofold. First, we seek to propose an experimental protocol that is usable as screening tool during training sessions and which is based on the execution of repeated vertical monopodalic jumps and single-leg squats. Second, we want to test the reliability of synthetic indices related to leg stability, load absorption and leg mobility, which can be considered both predictive risk factors and monitoring factors for the return-to-play time assessment after ACL reconstruction. As a further aim, the proposed experimental protocol and analysis methodology have been applied to a case study related to a female basketball player who suffered from ACL injury during the observational study.

## 2. Materials and Methods

### 2.1. Participants

Thirteen female athletes of the Italian Under 14 national basketball team (height = 171.4 ± 7.6 cm, body mass 65.4 ± 6.3 kg, age = 13 ± 1 years) participated in a fourteen-month observational study. One of the athletes, named hereinafter as athlete A, is present as a case study, since she suffered an ACL injury during an international basketball match less than two weeks after the first acquisition session. Specifically, medical staff reported an ACL rupture of the left knee caused by an incorrect landing phase after a monopodalic jump with a pivot-shift mechanism. The athlete underwent ACL reconstruction and her return-to-play was authorized after six months of rehabilitation. 

The remaining twelve athletes were included in an experimental protocol to both evaluate the reliability of the proposed synthetic indices and create reference values as a control group (CG). Athletes were included if they were in good health and without serious injuries in at least the last three years of activity, and if they trained regularly five days a week. 

The parents or tutors of each girl were asked to accept informed consent through a written document following a meeting in which the methods and objectives of the study were presented. The experimental protocol complied with the 1964 Helsinki Declaration.

### 2.2. Experimental Setup

Two devices were used for data acquisition during the experimental protocol, and both considered the reliability analysis and the case study: GyKo and Optogait. 

GyKo (Microgate S.r.l., Italy), or magneto-inertial measurements units (MIMUs), comprises 3D linear accelerometers, gyroscopes and magnetic sensors (±16 g, ±2000°/s and ±4800 μT of full-range scale, respectively) and it measures 3D linear acceleration, angular velocity and magnetic field vector. The sample frequency was set at 1000 Hz, which guaranteed the appropriate acquisition of highly dynamic tasks, such as jumps. The sensor was fixed using the semi-elastic belt supplied, as well as the specific magnetic support for the device, in order to reduce movement artifacts during tests. Specifically, it was fixed at the same height from the floor by the same operator to avoid errors in positioning. The same height also guaranteed the possibility of performing a comparison of stability indices related to players of different heights. Data acquired by the sensor were transmitted via Bluetooth to a personal computer and saved with Optogait software (version 1.12.1.0, Microgate S.r.l., Bolzano, Italy). The GyKo inertial sensor is commonly used for the evaluation of motor activities, such as jumping tests [31,32], muscle strength tests [33] and balance tests [32,34]. 

OptoGait is an optoelectronic device (Optogait, Microgate S.r.l, Bolzano, Italy, 2010) that consists of transmitting–receiving bars (length of 1 m) positioned above the ground. Each bar is equipped with ninety-six LED diodes, and they were synchronously associated with the GyKo in order to estimate the time of flight (FT) and the contact time (CT) during the jump tests. Four OptoGait bars were placed on the ground to create a square of 2 × 2 m^2^. The OptoGait system is mainly used in the analysis of human movements, especially in relation to gait [35,36] and jump gesture analyses [32]. Finally, two synchronized cameras (Logitech C920 HD) were used to acquire both sagittal and frontal plane images, and they were placed about 2.5 m from participants.

### 2.3. Experimental Protocol

Test sessions were held at the Istituto di Medicina e Scienza dello Sport (CONI) of Rome. Before each session, a 10-min warmup was performed by each athlete, with a focus on the lower limb joints. Specifically, mobility exercises and biking were required for each participant. The experimental protocol consisted of two tasks: a monopodalic countermovement jump (mCMJ) and a single-leg squat (SLS). At the beginning of each test, the subject was asked to remain stationary in a standing position, and players were asked to maintain an upright position for 10 s, with their arms at their sides and their feet shoulder-width apart and parallel to record data for the calibration of the sensors. Successively, the subjects were asked to execute the gesture, as detailed in the following paragraph, with both lower limbs. Each player first performed the tasks with their dominant leg, followed by their non-dominant leg.

#### 2.3.1. Monopodalic Countermovement Jump—mCMJ

**Figure 1:**
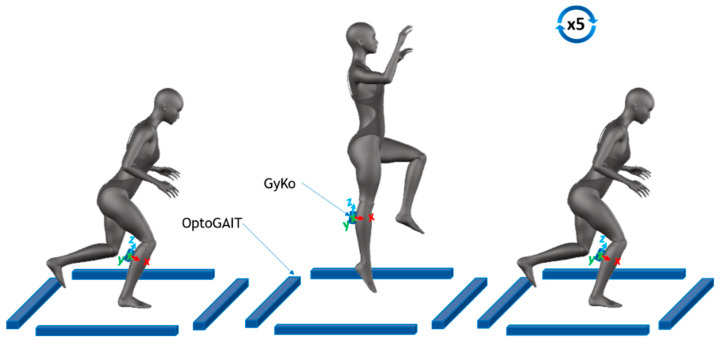
Monopodalic countermovement jump: sensor positioning and sequence of movements.

The mCMJ consisted of five consecutive monolateral vertical jumps with the arms free to move. Athletes were asked to reach the single-leg starting position with their foot shoulder-width apart and, after the start signal, to perform five maximal single-leg countermovement jumps (Figure 1). In the countermovement jumps, the subject started from a standing position with their feet shoulder-width apart and their arms free. Then, they performed a downward movement, which was immediately followed by a concentric phase. For this task, both GyKo and optoelectronic bars were used.

#### 2.3.2. Single-Leg Squats—SLS

**Figure 2:**
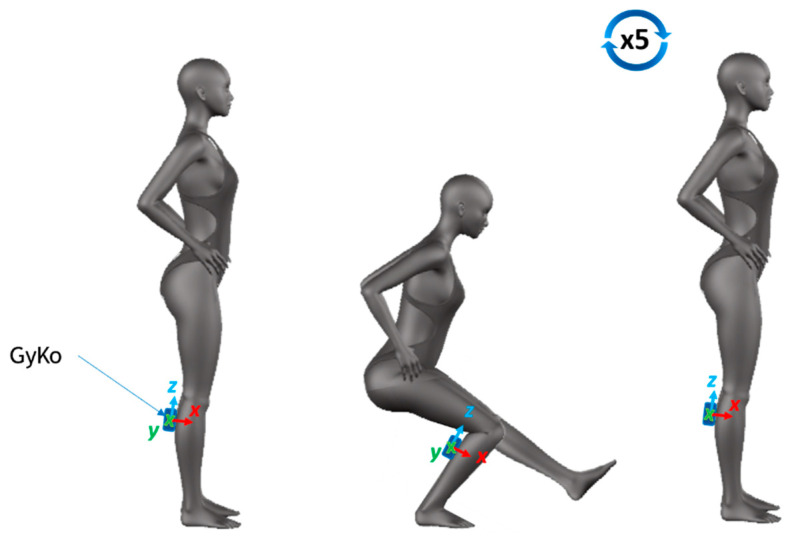
Single-leg squat: sensor positioning and sequence of movements.

The SLS is a squat movement performed on only one leg, and it avoids the requirement of a fixed angle that could constrain movement. The athletes started from a standing bipodal position and then passed on one leg while the other leg was lifted off the ground in front of the body. Having reached this position, subjects were asked to perform five consecutive squats with the standing limb. Subjects were instructed to maintain their arms with the hands on the hips and to descend as much as possible while maintaining position and balance [37] (Figure 2). For this task, only the GyKo was used; in fact, the task did not deal with contact and non-contact periods, and the use of the OptoGait was not necessary.

All participants were able to complete the entire protocol that lasted approximatively 20 min per athlete, including the sensorization phase. 

Athlete A completed four sessions: one before the ACL injury, and the following three sessions were carried out 6, 9 and 12 months after the operation; the average time between two consecutive sessions was 86 ± 5 days. These data were analyzed for the case study, and the sessions were hereinafter named PRE, POST1, POST2 and POST3. Instead, three sessions were carried out by the CG 3 months from each other; the average time between two consecutive sessions was 87 ± 4 days; the acquired data were used for the reliability analysis and to create reference values of the synthetic indices.

### 2.4. Data Analysis

Regarding the GyKo inertial sensor, the re-alignment of its axes with the absolute reference system was carried out by processing the data acquired during the static calibration for both tasks. The orientation of the sensor was computed by combining linear accelerations and angular velocities through a fusion algorithm based on the Mahony filter [38]. Three sets of parameters were successively calculated in order to evaluate the athletes’ performance in terms of leg stability, load absorption capability and leg mobility.

#### 2.4.1. Leg Stability

Concerning leg stability, parameters were calculated considering the orientation of the vertical axis ($\vec{z}$) of the sensor derived from the combination of the acceleration and angular velocity signals. The stability of the leg was analyzed considering the intersection between the axis related to the longest side of the sensor and the horizontal plane, corresponded to the floor, which was placed at 1 cm from the inertial system. The path covered by the intersection point was then analyzed using parameters typical of posturographic analysis [39]:

- Path length (PL), which is total length of the path in the plane.
- Path length in antero-posterior direction (PL_AP_).
- Path length in medio-lateral direction (PL_ML_).
- Ellipse area (EA), which is the minimum area of the bivariate confidence ellipse that contains at least 99% of the path.

Specifically, PL was computed by the sum of the distances between two consecutive points of the path covered by the intersection point (Equation (1)).

(1) $$PL=\overset{N-1}{\underset{n=1}{\sum }}{\left[{\left(AP\left[n+1\right]-AP\left[n\right]\right)}^{2}+{\left(ML\left[n+1\right]-ML\left[n\right]\right)}^{2}\right]}^{1/2}$$

where *N* represents the number of points of the path. The *PL* was also evaluated considering the antero-posterior (*PL_AP_*) and medio-lateral (*PL_ML_*) components one at a time. The *EA* was computed as in Equation (2):

(2) $$EA=2\pi F_{0.05\left[2,N-2\right]}{\left[s_{AP}^{2}s_{ML}^{2}-s_{APML}^{2}\right]}^{1/2}$$

where $F_{0.05\left[2,N-2\right]}$ is the *F* statistic at a 95% confidence level for a bivariate distribution with *N* points. $s_{AP}^{2}$ and $s_{ML}^{2}$ are the variance of the *AP* and *ML*, and $s_{APML}^{2}$ is the covariance.

**Figure 3:**
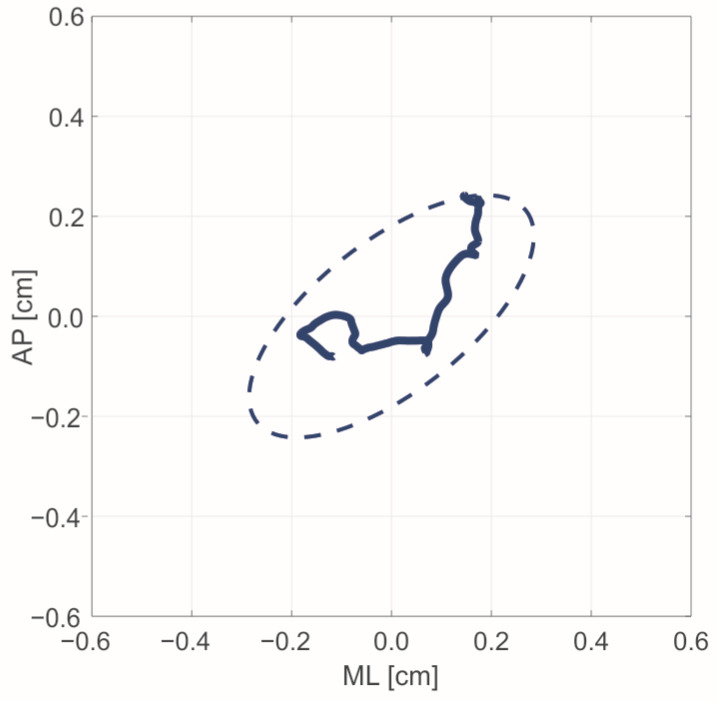
Example of path length (bold blue line) and confidence ellipse (dotted blue line) in the AP-ML plane.

A schematic example of *PL* and *EA* is reported in Figure 3.

**Figure 4:**
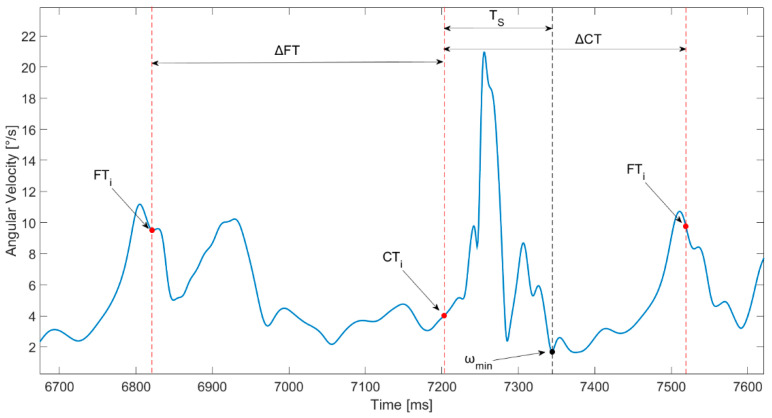
Identification of the stabilization time (Ts). ΔCT is contact time, ΔFT is flight time. CT_i_ and FT_i_ represent initial contact time and initial flight time phases, respectively.

For all the reported parameters, the greater the value, the higher the instability of the shank after landing; in fact, greater values of EA and PL indicate an increase in displacement [40]. We selected such indices, since it has already been demonstrated that the increase in instability immediately after landing can be considered as one of the main causes of ACL injury [11]. These parameters were computed for both tasks. Considering mCMJ, these parameters were normalized for the stabilization time (T_s_), which represented the time immediately after the landing phase that guaranteed the stabilization of the leg. Specifically, Ts was defined as the temporal distance between the initial contact time (CT) with the ground and the instant in which the leg could be considered stable, corresponding to the first minimum of the absolute value of the shank’s angular velocity, named as ω_min_ (Figure 4). The CT_i_ was detected by considering the on/off status of the LED in optoelectronic bars, which also allowed us to compute the duration of the flight time ΔFT; however, ω_min_ was automatically computed within the period of contact time ΔCT. 

**Figure 5:**
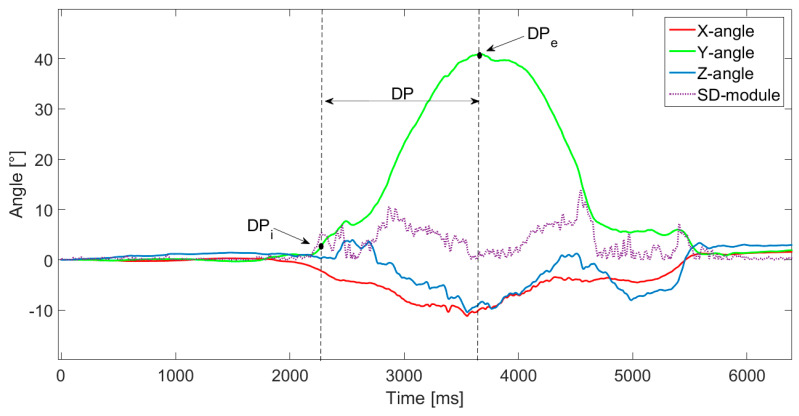
Identification of the descending phase (DP). DP_i_ is the initial instant of DP and DP_e_ is the end of DP.

Regarding the SLS, leg stability parameters were normalized for the descending phase time (T_DP_), which was the only analyzed phase, since it was characterized by a greater load on the knee joint [37]. To identify the initial instant of DP (DP_i_), the average value of the standard deviation (SD) of the module of the three angles on the X, Y and Z axes was first calculated in the first 2 s of each test individually per subject. Then, the initial instant was automatically identified by an ad hoc implemented algorithm as the one in which the average value of SD was overcome by the angle five times. The end point (DP_e_) corresponded to the maximum value reached by the angle around the Y axis (θ_ymax_), i.e., the one corresponding to knee flexion (Figure 5), as gathered from the GyKo data.

#### 2.4.2. Load Absorption Capability

The analysis of the capability to absorb a load after a jump was conducted by only considering data gathered during mCMJ. Specifically, shank linear acceleration was first divided into two components—vertical acceleration and acceleration on the horizontal plane xy, defined as the module of the two acceleration components x and y. Then, the root mean square was calculated for both components, vertical (RMS_z_) and along the plane xy (RMS_xy_), both expressed in m/s^2^. As for the stability indices, these parameters were finally normalized by dividing them by jump duration. Greater value of the RMS indicates poor capability for load absorption [41].

#### 2.4.3. Leg Mobility

The analysis of leg mobility was conducted only considering data gathered during SLS. Specifically, knee mobility was associated with the above-mentioned θ_ymax_. In particular, the lower the value of θ_ymax_, the lower the mobility [42].

### 2.5. Reliability Analysis

Reliability analysis was conducted by considering the data acquired during the three sessions performed by the CG to find the best reliable indices capable of evaluating changes in the subject affected by ACL injury. For each session, the average across the five jumps and squats for each parameter was calculated and then the inter-class correlation coefficient (ICC) among the three sessions based on absolute-agreement and two-way mixed-effects model was computed. Due to the symmetry the between right and left side, the analysis was only performed on the dominant leg, identified as the one used to kick a ball. ICC analysis allowed us to quantify inter-day reliability by understanding the effects induced by both the re-placement of the sensor on the shank and the daily changes in individual patterns when performing the technical gestures. Level of reliability was then classified according to the following ranges of ICC values: (i) poor reliability if ICC ranged from 0.00 to 0.39; (ii) fair reliability if ICC ranged from 0.40 to 0.59; (iii) good reliability if ICC ranged from 0.60 to 0.74; (iv) excellent reliability if ICC ranged from 0.75 to 0.99, as reported in [23]. The indices that showed at least good reliability, i.e., ICC values equal or greater than 0.60, were subsequently used for the case study.

### 2.6. Case Study—Statistical Analysis

Considering only the indices that passed the reliability analysis, the average values across all the participants in the control group were evaluated to create the reference values. Data gathered from the case study were processed and analyzed considering the PRE, POST1, POST2 and POST3 sessions individually. 

In order to identify the presence of statistical differences between data related to PRE and CG, a *t*-test was performed individually for each parameter. Any difference can lead to the identification of possible predictive factors. 

By only considering the parameters that were significant in the *t*-test in the PRE session, we performed a one-way ANOVA in order to identify the presence of statistical differences among the experimental sessions in the case study. Tests were conducted individually for each parameter. When statistical differences were found, a Bonferroni post hoc analysis for multiple comparisons was applied. Finally, each parameter related to each POST session was compared with the one associated with CG by performing a *t*-test in order to assess the effectiveness of the rehabilitation program on the injured leg and to identify indices that were able to monitor return-to-play time. The analyses were performed only on the injured leg; for the difference between the injured and non-injured leg, please refer to the preliminary study reported in [25].

For all the statistical analyses, the significance level was set at 0.05.

## 3. Results

### 3.1. Reliability Analysis

**Figure 6:**
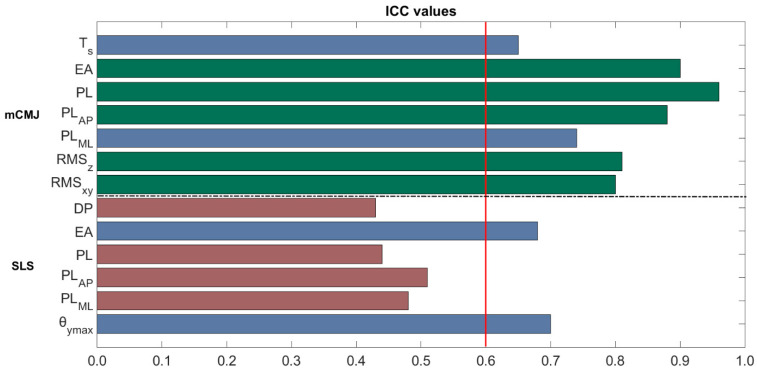
ICC values for all the computed parameters for both mCMJ and SLS task. Red, blue and green bars indicate fair, good and excellent reliability, respectively. The red vertical line represents the set threshold for acceptability, which is 0.60. The dotted black line separates the parameters related to the two motion tasks.

The ICC values for the mCMJ and SLS are reported in Figure 6.

By analyzing the mCMJ results, all parameters reached an ICC value greater than the acceptability threshold set at 0.60. Specifically, all parameters achieved results ranging from good-to-excellent reliability, with the maximum value obtained for the PL, i.e., equal to 0.96, and the minimum for the T_s_ (0.65). By moving to the SLS, only the EA and θ_ymax_ can be considered as reliable indices since they fall into the good reliability range, with values of 0.66 and 0.70, respectively. Conversely, all the other parameters always showed ICC values lower than 0.51, and they were not considered for the case study due to fair reliability.

### 3.2. Case Study

The results related to the comparisons between control group and athlete A for the PRE session are reported in Table 1 for both mCMJ and SLS. As already reported, the only parameters considered were the ones that showed an ICC ≥ 0.6.

By analyzing the results, PL, PL_AP_, RMS_z_ and RMS_xy_ related to the mCMJ task and the θ_ymax_ related to the SLS were statistically different between athlete A and the control group (*p*-value ranged from <0.01 to 0.02). More specifically, greater values extracted from the mCMH were obtained for athlete A compared to CG, as well as a lower θ_ymax_ in SLS. Conversely, no differences were found for Ts and for EA computed in both tasks.

**Figure 7:**
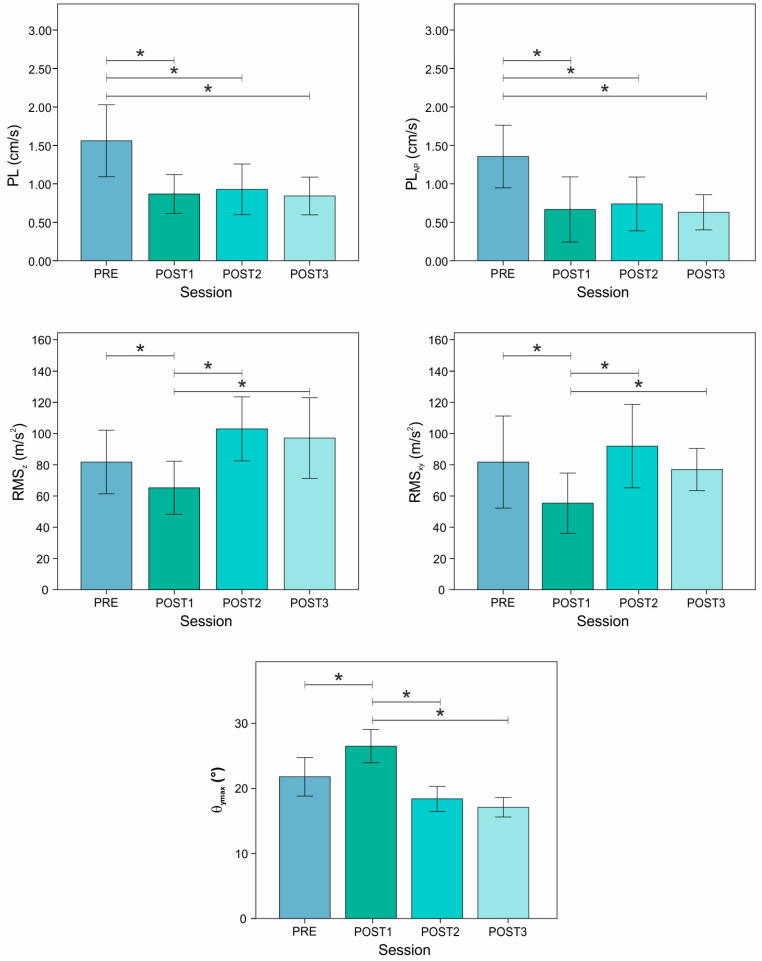
Means and standard deviations for each parameter related to each session. * indicates statistical difference between sessions.

Figure 7 shows the values for the selected indices related to the case study considering all experimental sessions, as well as statistical differences.

By analyzing the results, ANOVA tests exhibited statistical differences for all indices. Considering the leg stability indices, PRE value was found to be different in all the three POST sessions for both PL and PL_AP_ (*p*-value always <0.01). Conversely, no differences between the three POST sessions were found. Analyzing the load absorption capability, POST1 values were statistically different for both the PRE and the last two POST sessions for both RMS_z_ and RMS_xy_ (*p*-value ranging from <0.01 to 0.03). For the same parameters, no differences were found for PRE and the last two POST sessions. Furthermore, leg mobility showed the same behavior in load absorption parameters.

Finally, the results of the comparisons between POST and CG are depicted in Table 2. Considering leg stability, no differences with respect to CG were found for all three POST sessions. By moving to the indices associated with load absorption, both RMS_z_ and RMS_xy_ decreased in POST1, and no differences were found with respect to CG. Conversely, both parameters exhibited an increment of the mean value in POST2 and POST3, showing differences with respect to CG (*p* < 0.01). A similar trend was found for θ_ymax_.

## 4. Discussion

With the aim of investigating the identification of quantitative measurements able to identify ACL injury risk and monitor the time needed for injured athletes to return to play, we conducted a reliability analysis on parameters related to leg stability, load absorption and leg mobility when performing consecutive monopodalic jumps and single-leg squats. Then, we applied this innovative methodology to compare a control group of basketball players with an athlete who suffered from an ACL rupture during an observational study. 

### 4.1. Are the Proposed Indices Reliable for Evaluating Leg Stability, Leg Mobility and Load Absorption Capability?

Results permit us to assume that the proposed indices show a good-to-excellent reliability, considering all the parameters computed for the monopodalic jump tests. The reported inter-session reliability permits us to consider negligible the effects of both the sensor re-placement on the body segment and the intra-subject variability in motor tasks performed on different days. The negligible effects of the sensor re-placement can be ascribed to the application of the re-alignment operation as the first data processing step by using data acquired during static calibration, as well to the skilled operator performing the sensor placement. This finding demonstrates the high reliability, already shown in the literature, of both vertical bipodalic and monopodalic jumps [43]. Conversely, results recommend attention when considering parameters related to single-leg squat analysis, since only the angle and the ellipse area attained good reliability. Since the sensor re-placement and axes alignment procedures were performed both for monopodalic jump and leg squat analysis, we can speculate that the poor reliability of the squat indices is mainly due to the intra-subject variability in performing such a task over several days. This speculation confirmed the findings assessed by Van Der Straaten et al. [37], who showed that the kinematics related to the single-leg squat test, especially regarding the knee and the ankle joint, were characterized by poor reliability. One of the main causes that led to movement variability could be the absence of a clear endpoint for knee flexion, since athletes were instructed to reach the maximum possible knee flexion angle without losing balance [37]. In addition, in young and healthy populations, it has already been demonstrated that the achievement of a successful SLS can be the result of a combination of multiple movement strategies, also known as movement redundancy [44]. 

In conclusion, the parameters proposed here to analyze monopodalic jumps can be considered robust to both sensor re-placement and typical intra-day variability when analyzing jump tests; conversely, variability in the single-leg squat movement influences reliability outcomes. Finally, for all the indices that range from good-to-excellent reliability, we can affirm that any potential differences that occurred when comparing control group and injured athletes can be ascribed to ACL injury risk.

### 4.2. Should the Reliable Indices Be Monitored to Identify Athletes at Higher Injury Risk?

The analysis of the results obtained when comparing the control group with the data gathered from the athlete who suffered the ACL injury after the PRE session could highlight useful synthetic indices capable of discriminating higher injury risk. The statistically higher values of the path length, both in the plane and in an antero-posterior direction, related to athlete A, could be ascribed to an increment in the instability in the leg that is at higher risk when she lands after a jump. These outcomes are in line with previous studies, in which instability during landing or turning movements has been depicted as one of the main causes of ACL injury [11,45]. Conversely, the absence of statistical differences in the path length in a medio-lateral direction can be justified by the role of the tested ligament, which is mainly involved in the stabilization of the tibia or, more specifically of the knee, in the antero-posterior direction [46]. The higher values of the root mean square of the acceleration related to the injured athlete demonstrate a different load absorption strategy, suggesting an ineffective technique of load dissipation during landing. This outcome can lead to an increment in ACL injury risk, as also reported by [47]. Finally, leg mobility obtained during the single-leg squat test seems to be a further discriminating parameter for evaluating athletes with an ACL deficiency, confirming the outcomes reported by Markström et al. [46], who found a reduction in limb mobility during motor tasks in athletes with a higher injury risk. A lower value of θ_ymax_ can also lead to the inability of the hamstrings to restrain force placement on the ACL, causing potential risk of injury, as reported in [48]. To summarize, the results of the comparison between PRE sessions and CG allow us to encourage the analysis of leg stability after a monopodalic jump test, especially for considering the path length and the RMS of accelerations, as well as the evaluation of leg mobility during a single-leg squat test as a useful clinical tool to assess athletes at higher risk of anterior cruciate ligament injury. Thus, a periodic assessment of leg stability and load absorption, as well as joint mobility, should be adopted by athletic trainers to predict injury events early and implement specific training programs during coaching to reduce the risk. 

The outcomes obtained when comparing the POST sessions with the control group have to be taken into account in order to evaluate the effects of the rehabilitation program and to identify the correct return-to-play time. By analyzing the results related to POST1, we can assess that six months is a sufficient length of time for a complete functional recovery of the leg, as demonstrated by the absence of statistical differences between athlete A and the control group. These findings confirm the studies already proposed in literature that indicate six months as the right period to wait before returning to competition [49]. Several studies demonstrated a high rate of second injury in the two years after the initial trauma [50,51]. In this regard, our results showed a significant deterioration in motor performance in terms of load absorption capability and leg mobility both nine and twelve months after surgery; conversely, physiological behavior is maintained for leg stability. These outcomes suggest the necessity of implementing specific training programs for athletes with ACL deficiency, as characterized by leg mobility and the capacity for load absorption, as well as the usefulness of monitoring these parameters in order to predict the occurrence of a new ACL injury. 

## 5. Conclusions

The quantitative assessment of anterior cruciate ligament injury risk is still an untapped question. As such, we implemented an observational study over one year with basketball players who performed monopodalic countermovement jumps and single-leg squats. Data gathered from the experimental protocol were processed to obtain information associated with leg stability, load absorption capability and leg mobility. Here, we demonstrated the good and excellent inter-session reliability of some computed indices, and we applied this methodology on data gathered from an athlete who suffered from ACL injury and successive reconstruction during the observational study. The outcomes of the present study led to the identification of reliable quantitative metrics that should be used as a screening tool for both the assessment of athletes at higher injury risk and the evaluation of rehabilitation programs.

## Figures and Tables

**Table 1:** Mean (SD) of the parameters related to the mCMJ and SLS task for the PRE session for both control group (CG) and injured athlete (athlete A). * indicates statistical differences with respect CG.

	mCMJ							SLST	
	T_s_ [s]	EA [cm^2^/s]	PL [cm/s]	PL_AP_ [cm/s]	PL_ML_ [cm/s]	RMS_z_ [m/s^3^]	RMS_xy_ [m/s^3^]	EA [cm^2^/s]	θ_ymax_ [°]
***CG***	0.24 (0.06)	0.42 (0.12)	1.08 (0.15) *	0.75 (0.16) *	0.68 (0.07)	57.21 (7.99) *	48.69 (11.40) *	0.33 (0.11)	28.5 (2.6) *
**Athlete** **A**	0.22 (0.03)	0.30 (0.09)	1.56 (0.20) *	1.35 (0.20) *	0.58 (0.12)	81.73 (10.18) *	78.40 (14.15) *	0.32 (0.10)	21.8 (1.5) *

**Table 2:** Mean (SD) of the parameters related to the selected parameters for each POST session of the injured athlete (athlete A) and CG. * indicates statistical differences with respect to CG.

	PL [cm/s]	PL_AP_ [cm/s]	RMS_z_ [m/s^3^]	RMS_xy_ [m/s^3^]	θ_ymax_ [°]
***CG***	1.08 (0.15)	0.75 (0.16)	57.21 (7.99)	48.69 (11.40)	28.5 (2.6)
**POST1**	0.87 (0.13)	0.67 (0.21)	65.20 (8.41)	53.24 (9.20)	26.5 (1.3)
**POST2**	0.93 (0.16)	0.74 (0.17)	103.00 * (10.20)	88.25 * (12.81)	18.4 * (0.9)
**POST3**	0.84 (0.12)	0.63 (0.11)	97.11 * (12.86)	73.88 * (6.47)	17.1 * (0.7)

## Data Availability

Please contact the corresponding author.
